# To Assess the Outcomes Associated With the Use of Tranexamic Acid in the Open Fixation of Pelvic and Acetabular Fractures

**DOI:** 10.7759/cureus.38232

**Published:** 2023-04-27

**Authors:** Debkumar Chowdhury

**Affiliations:** 1 Department of Emergency Medicine, Manchester Royal Infirmary, Manchester, GBR

**Keywords:** geriatric injuries, intravenous tranexamic acid, open acetabular fracture, open pelvic fracture, trauma

## Abstract

There is a growing knowledge base for the use of antifibrinolytic therapy in trauma and orthopaedic surgery. The mechanism of action of tranexamic acid (TXA) is through the inhibition of fibrinolysis. The role of TXA in hip fractures has been researched extensively. However, the research has been limited to the cases of pelvic and acetabular fractures. This systematic review aimed to examine the effect of TXA on patients undergoing open pelvic and acetabular fracture surgery. The primary goal of the study was to examine the estimated blood loss (EBL) and blood transfusion rates in patients who received TXA versus those who did not receive TXA. The secondary goal was to examine the rates of deep venous thrombosis (DVT). A literature search was carried out using PubMed, Medline and the Cochrane database. The selection criteria for the systematic review were studies investigating intravenous TXA in the form of randomised controlled trials (RCTs), as well as cohort studies. Five studies were included in the systematic review with 625 total patients. The EBL between the two groups was noted to be 661 mL in the control group and 850 mL in the TXA (*p*=0.49). There was a marginally lower number of units transfused in the control group vs the TXA group (1.9 vs 2.2) (p=0.27). The rates of transfusion in the TXA group were 29% TXA whilst, in the control group, it was 31% (p=0.13). The overall incidence of DVT was 2.8% in the TXA group and 1.7% in the control group (p=0.097).

## Introduction and background

There is a growing knowledge base for the use of antifibrinolytic therapy in trauma and orthopaedic surgery. The mechanism of the action of tranexamic acid (TXA) is through inhibition of fibrinolysis with the prevention of the interaction between plasmin and the fibrin binding site [1]. Orthopaedic trauma surgeries are associated with higher rates of blood loss, with the need for blood transfusions. The need for blood transfusion is associated with an increased risk of transfusion-related complications as well as increased cost and length of hospital stay [2,3]. The rationale for the use of the antifibrinolytic agent, TXA, is to limit these undesirable effects associated with blood transfusion.

The beneficial effect of TXA in joint arthroplasties has been well demonstrated to reduce blood loss without increased risk of thromboembolism [4-6]. In the context of trauma, a large meta-analysis carried out by Kerr et al. demonstrated that with TXA the transfusion requirement was reduced by 30-33% [3]. A large systematic review and meta-analysis done by Karl et al. demonstrated that the use of TXA resulted in a significant decrease in one-month mortality when compared to the cohort in bleeding trauma patients [7]. In this study, the impact of TXA on thromboembolism could not be analysed due to the heterogeneity between the groups.

However, the evidence for the benefit of TXA in acetabular and pelvic fracture fixation is limited. The reduction in transfusion requirement and estimated blood loss (EBL) with the use of intravenous TXA has not been uniformly noted across the studies. The retrospective cohort study by Wadhwa et al. demonstrated with intravenous TXA, there was no improvement in the EBL or transfusion requirements [8]. Acetabular and pelvic fracture fixation is associated with longer operative times when compared to hip fracture surgery [8].

Therefore, a systematic review was carried out after establishing the need to evaluate the literature on the role of TXA in open acetabular and pelvic fractures.

The overall objective of this systematic review was to evaluate the use of TXA in pelvic and acetabular fracture surgery. The primary goal was to compare the transfusion rates and EBL in patients receiving TXA versus those not receiving TXA. The secondary goal was to compare the rates of deep venous thrombosis (DVT) with the use of TXA.

The study hypothesises that intravenous TXA is associated with reduced intra-operative blood loss and transfusion rates without any increase in the rates of DVT.

This article was previously posted to the Research Square preprint server on March 2, 2023.

Methodology

**Figure 1:**
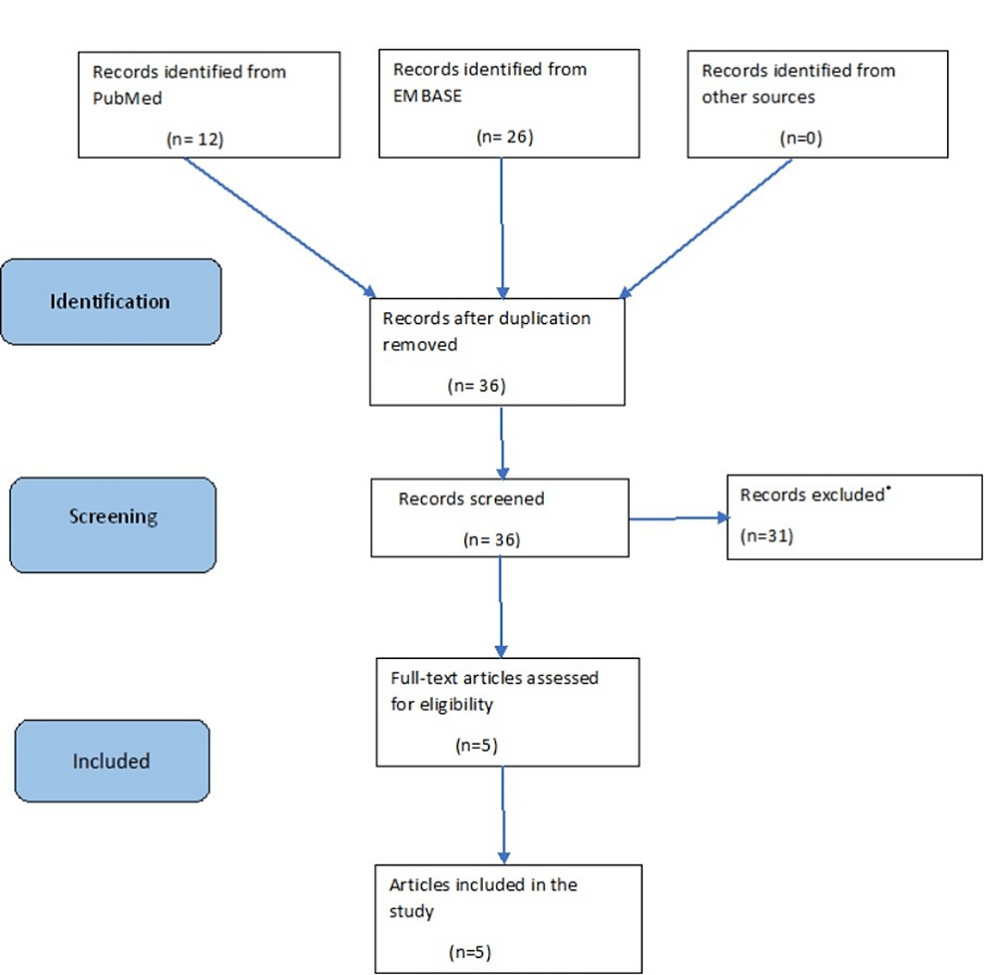
PRISMA 2020 flow diagram Results excluded *- Studies that did not meet the inclusion criteria - both the estimated blood loss/transfusion rates and the rates of deep venous thrombosis. PRISMA: Preferred Reporting Items for Systematic Reviews and Meta-Analyses

A systematic review was carried out using the studies from two separate databases, PubMed and Medline using the Preferred Reporting Items for Systematic Reviews and Meta-Analyses (PRISMA) checklist (see Figure 1) [9]. The Cochrane database was also searched to ascertain if any similar studies had previously been undertaken. The systematic review was registered on the PROSPERO international prospective register of systematic review (registration number CRD42022316445) on March 23, 2022. The individual steps of identification, screening, eligibility and inclusion criteria have been included in the PRISMA diagram.

Data analysis

The outcomes for this systematic review were mean EBL, number of units of blood transfused, transfusion rates and rates of DVT. In addition, the operative times and the surgical approaches were also evaluated. A primary meta-analysis could not be undertaken due to the heterogeneity of the study design of the individual studies. Statistical analyses were carried out using Statistical Product and Service Solutions (SPSS) (IBM SPSS Statistics for Windows, Version 27, Armonk, NY) [10]. For the mean results p-values were generated using the aforementioned software. Results with a p<0.05 were considered to be statistically significant. The data was subsequently entered into spreadsheets created using the Excel 2007 programme by Microsoft (Microsoft Corporation, Redmond, Washington, USA) [11]. A comparative analysis of the EBL was subsequently demonstrated in the form of bar graphs for better visual representation.

Study eligibility criteria

The study eligibility criteria were defined using the Population, Intervention, Control, Outcome (PICO) framework as can be seen in Table 1 [12].

**Table 1:** The PICO process PICO: Population, Intervention, Control, Outcome

Items	Description
Population	Adult patients undergoing open operative fixation of pelvic and acetabular fractures.
Intervention	Tranexamic acid (15-18mg/kg) both in the pre-operative and intra-operative phases.
Control	Dependent on the study design.
Outcome	Primary outcome - estimated blood loss (EBL) and the transfusion rates in the individual studies. A secondary outcome of interest - is rates of deep venous thrombosis.

Search strategy and data sources

The data was collected using search criteria using Medical Subject Headings (MeSH) terms as well as Boolean operators to maximise the number of suitable studies [13]. The following search strategy was used as demonstrated below in Table 2.

**Table 2:** Search strategy TXA: tranexamic acid

Items	Descriptors
Search Terms	‘Bleeding’, ‘Haemorrhage’, ‘Tranexamic acid’, ‘Acetabular fractures’, ‘Pelvic fractures’, ‘Open fixation’, ‘Deep venous thrombosis'
Boolean operators	(TXA OR Tranexamic Acid OR antifibrinolytic) (Bleeding OR haemorrhage OR haemorrhage OR blood loss) (Pelvic fracture OR Acetabular fracture) AND (TXA AND blood loss AND pelvic fractures) NOT to exclude hip fractures (TXA AND blood loss AND pelvic fractures NOT hip fracture)

Randomised controlled trials (RCTs) and cohort studies (prospective and retrospective) on human subjects were included from 2010 to 2022 in the analysis. The search for studies was carried out in the English language.

Study selection and data collection

A clear exclusion criterion has been adhered to ensure uniformity in the data collection. The inclusion and exclusion have been tabulated as demonstrated below in Table 3.

**Table 3:** Inclusion and exclusion criteria RCTs: randomised controlled trials

Inclusion criteria	Exclusion criteria
Studies (RCTs and cohort) studying direct comparison between tranexamic acid and a placebo. Studies without a direct comparison were also included. Both retrospective and prospective cohort studies were included.	Additional intercurrent procedures for other injuries.
Reported estimated blood loss and transfusion rates.	Disorders of coagulation or medications that would affect coagulation.
Reported the incidence of venous thrombosis.	Concurrent injuries would be a contraindication to venous thrombi-embolism prophylaxis.
	Perioperative mortality and other causes of loss to follow-up.
	Closed fracture fixation

Studies (RCTs and cohort) studying direct comparison between TXA and a placebo. Studies without a direct comparison were also included. Both retrospective and prospective cohort studies were included.

Data collection and data items collected

Following the review of the included studies, the data was collected as demonstrated in Table 4.

**Table 4:** Data collected from the individual studies BMI: body mass index; EBL: estimated blood loss

Item number	Description of data items collected
1	Characteristics of the study - the author, year, location title of the study, design, the total number of patients (further subdivision into treatment and placebo groups), the transfusion threshold and the tranexamic acid dose protocol used.
2	Average cumulative pooled data from the studies - age, gender, BMI, number of units of blood transfused, operative time and EBL.
3	The rates of blood transfusion in the individual studies that have reported this data.
4	The rates of deep venous thrombosis in individual studies.

Quality assessment and risk of bias assessments

The risk of bias assessment was carried out using the Cochrane Risk of Bias Tool for RCTs [14]. RCTs were assessed for quality and risk of bias using the Grading of Recommendations, Assessment, Development, and Evaluations (GRADE) assessment tool as can be seen in Table 5 [15]. Non-randomised controlled trials (non-RCTs) were assessed using the Methodological Index for Non-Randomised Studies (MINORS) tool as can be seen in Table 6 [16]. The individual studies were assessed for random sequence generation, allocation concealment as well as assessing for various biases. Utilising the GRADE assessment tool, the RCTs were noted to be well-designed with a low risk of individual biases as demonstrated in Table 5. For the non-RCTs, utilising the MINORS assessment tool, it was noted that individual elements were missing in the studies conducted by Cohen et al. [17] and Gumustas et al. [18].

**Table 5:** GRADE assessment for randomised controlled trials GRADE: Grading of Recommendations, Assessment, Development, and Evaluations

Study	Random sequence generation	Allocation concealment	Performance bias	Detection bias	Attrition bias	Reporting bias	Other bias
Lack et al., 2017 [19]	Low Risk	Low Risk	Low Risk	Low Risk	Low Risk	Low Risk	Low Risk
Spitler et al., 2019 [20]	Low Risk	Low Risk	Low Risk	Low Risk	Low Risk	Low Risk	Low Risk

**Table 6:** MINORS assessment tool for the non-randomised controlled trials Loss to f/u: loss to follow up; MINORS: Methodological Index for Non-Randomised Studies

MINORS assessment Tool												
Study	Aim	Inclusion of consecutive patients	Data collection	Endpoints	Unbiased assessment	Follow up	Loss to f/u	Study size	Adequate control	Contemporary	Baseline	Statistical analyses
Cohen et al., 2020 [17]	Yes	Yes	Yes	Yes	N/A	Yes	No	N/A	Yes	Yes	Yes	Yes
Gumustas et al., 2022 [18]	Yes	Yes	Yes	Yes	N/A	Yes	No	N/A	N/A	N/A	N/A	Yes
Wadhwa et al., 2022 [8]	Yes	Yes	Yes	Yes	Yes	Yes	No	Yes	Yes	Yes	Yes	Yes

Ethical statement

As the study was a systematic review, ethical approval was not required.

Results

Quality assessments of the individual studies were carried out, and the RCTs were noted as of high quality with the remainder of the studies being of intermediate quality as can be seen in Table 5 and Table 6.

From all the studies there were 625 patients included in the five studies in this systematic review and have been reported using the PRISMA checklist (9) as demonstrated in Figure 1 and Table 7. Two of which were RCTs, one prospective and two retrospective cohort studies.

**Table 7:** Summary of the studies included in the systematic review N: number of patients; Mean EBL (mL): mean estimated blood loss (millilitres); DVT: deep venous thrombosis; Units T/F: units transfused

Author, location	Design	N	Cohort	Mean EBL (mL)	DVT	Units T/F
Lack et al., 2017 [19], USA	Prospective, Multicentre, Randomised Controlled Trial	88	TXA group - 42	TXA - 727.6	TXA - 1	TXA - 2.65
			Placebo - 46	Placebo - 560.1	Placebo - 0	Placebo - 2.36
Spitter et al., 2019 [20], USA	Prospective, Multicentre, Randomised Controlled Trial	93	TXA group - 47	TXA - 805	TXA - 2	TXA - 1.53
							Placebo - 46	Placebo - 655	Placebo - 1	Placebo - 1.19
							Cohen-Levy et al., 2020 [17], USA	Retrospective cohort study	159	TXA group - 159	TXA - N/A	DVT - 10	TXA - N/A
			Gumustas et al., 2022 [18], Turkey	Prospective cohort study	73	TXA group - 73								TXA - 1137	DVT - 0	TXA - 2.1
Wadhwa et al., 2022 [8], USA	Retrospective cohort study	305					TXA group - 89	TXA - 729.8	TXA - 1	TXA - 2.40						
			Placebo - 216	Placebo - 768.1	Placebo - 6	Placebo - 2.14

Primary outcome

**Figure 2:**
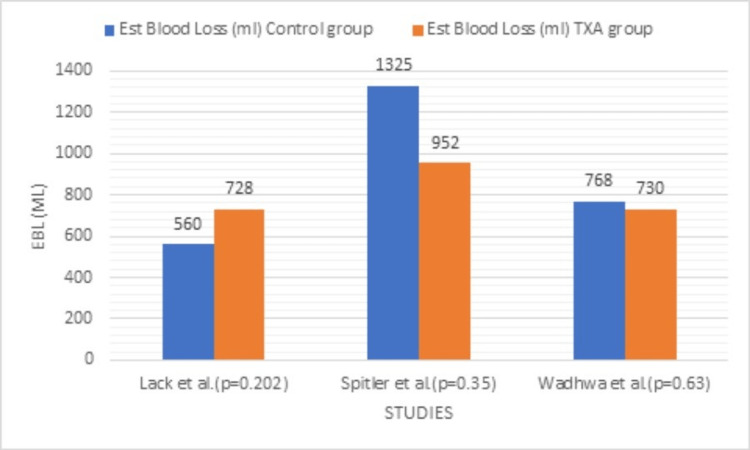
Comparative analyses of estimated blood loss in the different studies Lack et al., 2017 [19], Spitler et al., 2019 [20], Wadhwa et al., 2022 [8] Est Blood Loss (mL): estimated blood loss (millilitres)

In the context of EBL, in the study by Cohen-Levy et al. [17], the EBL was not explicitly documented. In the study by Gumustas et al. [18], a comparative analysis could not be undertaken as there was no control group. In the analysis of the remaining three studies, the mean EBL in the TXA group was noted to be higher when compared to the placebo group (mean - 850mL vs 661mL) (p=0.49) as demonstrated in Table 8 and Figure 2. However, the EBL volumes within the individual studies did not demonstrate statistical significance. In terms of other patient characteristics, the age and gender ratios were comparable between the TXA and the control groups. The average body mass index (BMI) was also comparable between the two groups.

**Table 8:** Average cumulative pooled data from the studies TXA: tranexamic acid; BMI: body mass index; EBL: estimated blood loss (millilitres)

	TXA group (250)	Control (No TXA) (375)
Age (years)	46.9	45.8
Male	183 (73%)	272 (72%)
Female	67 (27%)	103 (27%)
BMI (kg/m^2^)	31.3	31.7
Blood transfused (number of units)	2.2	1.9
Operative time (min)	222	250
EBL (mL)	850	661

A discrepancy was noted in terms of the transfusion thresholds as well as the TXA dosing regimen as demonstrated in Table 9. Lack et al. [19] had a lower transfusion threshold of haemoglobin (Hb)<7g/dL whilst Wadhwa et al. [8] there was no predefined transfusion target. The study by Spitler et al. [20] had a transfusion threshold of Hb<8g/dL. In terms of the TXA dosing regimen, this was different in each of the studies. The total amount of TXA administered also varied in each of the studies. The study by Cohen et al. [17] had both the transfusion threshold and TXA dose varied depending on the treating surgeon and anaesthetist.

**Table 9:** Characteristics of the studies highlighting the transfusion thresholds Hb: haemoglobin; TXA: tranexamic acid

Study	Type of study	Treatment group	Control group	Transfusion threshold	TXA dose
Lack et al., 2017 [19]	Randomised controlled trial	42	46	Hb<7g/dL	10mg/kg pre-operative, 10mg/kg over 4 hours
Spitler et al., 2019 [20]	Randomised controlled trial	47	46	Hb<8g/dL	15mg/kg preoperative, 15mg/kg over 3 hours afterwards
Cohen-Levy et al., 2020 [17]	Retrospective cohort study	159	0	Discretion of the treating surgeon and anaesthetist	No set protocol for dosing
Gumustas et al., 2022 [18]	Prospective cohort study	73	0	Hb>10g/dL	1g Pre-op
Wadhwa et al., 2022 [8]	Retrospective cohort study	89	216	Discretion of the treating team	1g at time of incision, 1g afterwards

A marginally higher average number of units were transfused in the TXA group in comparison to the control group (2.2 Units vs 1.9 Units) (p=0.27). The results have been highlighted in Table 7. The rates of transfusion in the TXA group were 29% TXA whilst, in the control group, it was 31% (p=0.13) as highlighted in Table 10. Studies by Spitler et al. [20] and Wadhwa et al. [8] had a lower rate of blood transfusion in the TXA group in comparison to the control group. However, it can be seen that in the individual studies, there was no statistical significance between the transfusion rates.

**Table 10:** Rates of transfusion in the different studies TXA: tranexamic acid

Study	Numbers in treatment group	Numbers in control group	Transfusion rates (%)		P value
			TXA	Control	
Lack et al., 2017 [19]	42	46	21 (50)	15 (32)	0.097
Spitler et al., 2019 [20]	47	46	8 (17)	14 (30)	0.13
Cohen-Levy et al., 2020 [17]	159	0	82 (51.6)	0	N/A
Gumustas et al., 2022 [18]	73	0	15 (21)	0	N/A
Wadhwa et al., 2022 [8]	89	216	20 (22.5)	64 (29.6)	0.2

Secondary outcome

The average incidence of DVT was 2.8% in the TXA group and 1.7% in the control group (p=0.097) as demonstrated in Table 11. The study by Gumustas et al. [18] did not have any reported cases of DVT. The study by Wadhwa et al. [8] had a higher incidence of DVT in the control group in comparison to the TXA group. In the remainder of the studies, there was a higher incidence of DVT in the TXA group. However, the rates of DVT did not reach statistical significance in the individual studies.

**Table 11:** DVT rates in the different studies DVT: deep venous thrombosis; TXA: tranexamic acid

Study	Type of study	Numbers in treatment group	Numbers in control group	DVT rates (%)		P value
				TXA	Control	
Lack et al., 2017 [19]	Randomised controlled trial	42	46	1 (2.4)	0	1
Spitler et al., 2019 [20]	Randomised controlled trial	47	46	2 (4.3)	1 (2.2)	0.45
Cohen-Levy et al., 2017 [17]	Retrospective cohort study	159	0	10 (6.3)	0	0.75
Gumutas et al., 2022 [18]	Prospective cohort study	73	0	0	0	N/A
Wadhwa et al., 2022 [8]	Retrospective cohort study	89	216	1 (1.1)	6 (2.8)	0.68

## Review

Discussion

The results of this review show that TXA did not reduce the average number of units transfused, the EBL and the overall incidence of DVT in comparison to the control group. A marginal reduction in the rates of transfusion was demonstrated with the use of TXA, however, this was not demonstrated to reach statistical significance. The results of this review highlight that whilst TXA does not increase the incidence of DVT, its role in limiting EBL is limited with no significant improvement in transfusion rates with its use.

Venous thrombosis

The rates of DVT from this systematic review are reflective of the pre-existing literature that the use of TXA does not lead to an increased risk of venous thrombosis [21]. The results from this systematic review are important to the clinician to allay any fears of increased risk of DVT with the use of TXA.

In the study by Gumustas et al. [18], there were no reported cases of DVT. In the study by Wadhwa et al. [8], there was a proportionally higher rate of DVT in the control group compared to TXA (2.8% vs 1.1%) however this was not statistically significant. In two of the studies Lack et al. [19], and Spitler et al. [20] there was a greater proportion of patients in the TXA group that had DVT, however, this did not reach statistical significance.

Research by Levy-Cohen et al. [17] reported a higher overall rate of DVT in the TXA group compared to the other studies. However, the overall results highlighted that the group that withheld preoperative chemoprophylaxis in combination with intraoperative administration of TXA did not increase the rates of DVT.

TXA dose and timing

The dose and timing of TXA were varied across the groups with the protocol being different in each of the studies. The optimal timing and appropriate dosage of TXA for the most effective haemorrhage control to achieve a subsequent reduction in the rates of blood transfusions is a matter of debate [22]. Research by Sershon et al. [23] highlighted that there were no differences in EBL or transfusion rates regarding the dose and timing of TXA in revision total hip arthroplasty. However, similar comparative research is not available for pelvic and acetabular fracture surgery.

Pelvic and acetabular operations are more complex and operative times are longer when compared to hip fracture surgery which could signify a longer bleeding risk [8]. As a result, there remains a concern about whether earlier administration of TXA may be beneficial to reduce the risk of bleeding. Research by Wadhwa et al. [8] did involve a sizeable cohort of patients that received TXA. Although a comparative analysis was not possible with this study, the results could be cumulative to the overall effect of TXA. Incorporating the results from the study by Wadhwa et al. [8] added to the knowledge base regarding the role of TXA in acetabular fracture surgery.

Transfusion thresholds

Research by Spitler et al. [20] reported the transfusion threshold to be Hb<8g/dL [19,20]. The threshold was noted to be variable in the remaining studies. Research by Wadhwa et al. [9] found the transfusion threshold was at the discretion of the treating clinician. Research by Lack et al. [19] reported a lower transfusion threshold of Hb<7g/dL was utilised. Having a lower transfusion threshold may signify that a more permissible lower level of Hb would be considered before transfusion. A restrictive target of Hb 7-8 in hip fractures has been demonstrated not to lead to an increase in mortality when compared to a more liberal approach [24].

The average age of the patients in this systematic review was 46 years, comparable to the existing literature, which may be lower than the average age of patients sustaining hip fractures [25]. Although the underlying co-morbidities have not been stated in the individual studies, the incidence of cardiovascular disease may be considered lower in this group in comparison to a similar study for hip fractures [26]. Patients in this systematic review may therefore tolerate a lower permissible Hb level. The results would however need to be evaluated in the context of acetabular and pelvic fractures. The requirement for blood transfusion has been identified as an independent predictor of mortality in the context of traumatic shock [27].

Limitations

The main limitation that was encountered in the conduct of this systematic review is a dearth of RCTs highlighting the required dataset. As a result, there was a need for the inclusion of poor-quality studies including non-RCTs where a direct comparison between TXA and placebo could not be possible. There has been much focused research in the domain of hip fractures with a limited number of studies highlighting pelvic and acetabular fractures open reduction and internal fixation [2].

In addition to the above, there was noted to be heterogeneity within the available studies included in the systematic review. As a result of the heterogeneity, a meta-analysis could not be carried out. As aforementioned previously, there was heterogeneity about the transfusion targets, it is unclear if this would have any impact on the number of units transfused. The rationale for the inclusion of the study by Gumustas et al. [18] was that this was a recent prospective study with a standardised dose of TXA administration.

The findings report variability in the timing of the administration of TXA as well as dosing, few of the studies had administration pre-operatively and subsequently intra-operatively. This could potentially lead to treatment bias. Some of the studies chose between 10 and 15mg/kg while others had a fixed dose of 1g. The dose for optimal effectiveness needs to be further evaluated through future study protocols. Two important factors that need to be considered are the amount of TXA administered and the timing of administration.

Although there has been a recent systematic review comparing the effect of TXA in pelvic and acetabular fractures, this systematic review includes a larger number of studies with larger patient populations [28].

## Conclusions

The risk of bias and measures to determine the quality of assessment was also noted to be missing from the previously conducted systematic review which has been addressed here. This systematic review concludes that TXA has a limited role in limiting the EBL without a significant overall benefit in reducing the need for blood transfusion. The average rates of DVT were noted to be higher in the TXA group in comparison to the control group, however, the rates of DVT did not reach statistical significance in the individual studies. Through this systematic review, it has been established that there is a need for large well-designed prospective RCTs that would evaluate the true impact of TXA further and reach statistical significance with standardisation of the dose of TXA.
